# Reference Gene Validation in the Brain Regions of Young Rats after Pentylenetetrazole-Induced Seizures

**DOI:** 10.3390/biomedicines8080239

**Published:** 2020-07-23

**Authors:** Alexander P. Schwarz, Anna A. Kovalenko, Daria A. Malygina, Tatiana Y. Postnikova, Olga E. Zubareva, Aleksey V. Zaitsev

**Affiliations:** 1 Sechenov Institute of Evolutionary Physiology and Biochemistry of RAS, 44, Toreza Prospekt, Saint Petersburg 194223, Russia; aleksandr.pavlovich.schwarz@gmail.com (A.P.S.); kovalenko_0911@mail.ru (A.A.K.); malygina.darja@yandex.ru (D.A.M.); tapost2@mail.ru (T.Y.P.); zubarevaoe@mail.ru (O.E.Z.); 2 Almazov National Medical Research Centre, Institute of Experimental Medicine, 2 Akkuratova Street, Saint Petersburg 197341, Russia

**Keywords:** reference gene stability, rat brain, pentylenetetrazole, seizure model, gene expression analysis, qRT-PCR

## Abstract

Reverse transcription followed by quantitative polymerase chain reaction (qRT-PCR) is a powerful and commonly used tool for gene expression analysis. It requires the right choice of stably expressed reference genes for accurate normalization. In this work, we aimed to select the optimal reference genes for qRT-PCR normalization within different brain areas during the first week following pentylenetetrazole-induced seizures in immature (P20–22) Wistar rats. We have tested the expression stability of a panel of nine housekeeping genes: *Actb, Gapdh, B2m*, *Rpl13a, Sdha, Ppia*, *Hprt1, Pgk1,* and *Ywhaz.* Based on geometric averaging of ranks obtained by four common algorithms (geNorm, NormFinder, BestKeeper, Comparative Delta-Ct), we found that the stability of tested reference genes varied significantly between different brain regions. The expression of the tested panel of genes was very stable within the medial prefrontal and temporal cortex, and the dorsal hippocampus. However, within the ventral hippocampus, the entorhinal cortex and amygdala expression levels of most of the tested genes were not steady. The data revealed that in the pentylenetetrazole-induced seizure model in juvenile rats, *Pgk1*, *Ppia*, and *B2m* expression are the most stable within the medial prefrontal cortex; *Ppia*, *Rpl13a*, and *Sdha* within the temporal cortex; *Pgk1*, *Ppia*, and *Rpl13a* within the entorhinal cortex; *Gapdh*, *Ppia*, and *Pgk1* within the dorsal hippocampus; *Rpl13a*, *Sdha,* and *Ppia* within the ventral hippocampus; and *Sdha*, *Pgk1*, and *Ppia* within the amygdala. Our data indicate the need for a differential selection of reference genes across brain regions, including the dorsal and ventral hippocampus.

## 1. Introduction

Reverse transcription followed by quantitative polymerase chain reaction (qRT-PCR) is a powerful tool for measuring relative gene expression in biomedical research. However, precise measurement requires valid reference genes for normalization of the gene of interest’s expression level [1]. The selection of unstable housekeeping genes as references could affect accurate quantification, leading to inconsistent results. Normalization using unstable reference genes could hide changes in target mRNA expression or find irrelevant effects of experimental treatments [2,3,4,5,6,7,8]. In the rat valproic acid model of autism spectrum disorders, changes of *Pgrn1* mRNA expression in the hippocampus and cortex can have opposite directions depending on reference gene choice [3]. Reference gene expression stability varies depending on experimental settings, tissue type, and brain region [9]. Several algorithms have been developed to evaluate reference gene expression stability; the most popular methods are geNorm [10], NormFinder [11], BestKeeper [12], and the comparative Delta-Ct approach [13]. Despite differences in the calculations used, these methods are all based on the idea that expressions of stable reference genes relative to other candidate genes should be the least variable across experimental samples. Different algorithms provide slightly different results [2,14,15]. To escape that bias, this paper uses a novel approach by calculating a comprehensive reference gene stability ranking based on four algorithms [3,7,15,16,17,18,19].

Among different reference genes, *Gapdh*, *Actb*, *Ppia*, *B2m*, *Hprt1*, *Ywhaz*, *Rpl13a*, *Pgk1*, and *Sdha* are widely used in RT-qPCR experiments on brain tissue or brain-derived cell lines in laboratory rats. These genes show high stability in different experimental settings [9]. Their encoded proteins participate in different cell functions: glycolysis (*Gapdh*, *Pgk1*), electron transport chain (*Sdha*), antigen presentation (*B2m*), translation (*Rpl13a*), purine metabolism (*Hprt1*), cell signaling (*Ywhaz)*, protein folding *(Ppia)*, and cytoskeleton building *(Actb).*

In the present work, we aimed to evaluate the expression stability of those nine housekeeping genes. We then selected the most suitable reference genes for RT-qPCR analysis within different brain areas in the pentylenetetrazole (PTZ) seizure model in immature rats. The PTZ model of seizures is widely used to investigate the mechanism of neuropsychiatric disturbances caused by severe childhood seizures [20,21,22,23,24]. Immature rats are more sensitive to PTZ compared to adults, and PTZ induces prolonged tonic-clonic seizures [25]. PTZ-induced seizures are characterized by massive activation of a broad spectrum of cortical and subcortical brain areas, especially in young rats, leading to delayed changes in synaptic plasticity and behavior [21,22,24,25,26,27].

## 2. Materials and Methods

### 2.1. PTZ Model of Seizures

This study used 20–22-day-old male Wistar rats (35–40 g). All the experiments were carried out under the Guidelines on the Treatment of Laboratory Animals effective at the Sechenov Institute of Evolutionary Physiology and Biochemistry of the Russian Academy of Sciences (Ethical permit number 13-k-a, 15 February 2018). These guidelines comply with EU Directive 2010/63/EU for animal experiments.

**Figure 1:**
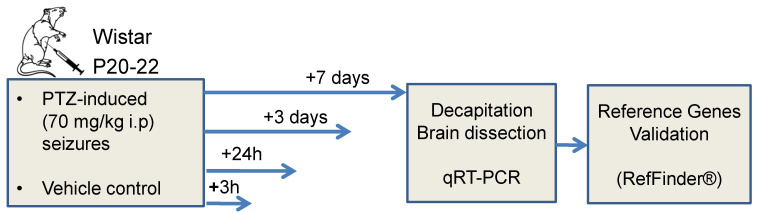
Outline of animal experiments. Wistar rats were injected with PTZ (70 mg/kg) or saline vehicle (control) intraperitoneally at postnatal day (P) 20–22. *n* = 4–7 per group.

Acute seizures were induced by PTZ administration (70 mg/kg in saline; intraperitoneal (i.p.), Sigma Aldrich, St. Louis, MO, USA). Approximately 80% of treated rats had generalized tonic-clonic seizures lasting more than 45 min, i.e., exhibited status epilepticus. Only those animals were used in the experiments. The control rats were injected with saline. Gene expression level was measured 3 h and 1, 3, and 7 days after the administration of PTZ or saline (*n* = 4–7 animals per group). The total number of animals used in RT-qPCR experiment was 47 (i.e., limited to one 96-well PCR plate). Figure 1 summarizes the experimental outline.

### 2.2. mRNA Extraction and cDNA Synthesis

**Figure 2:**
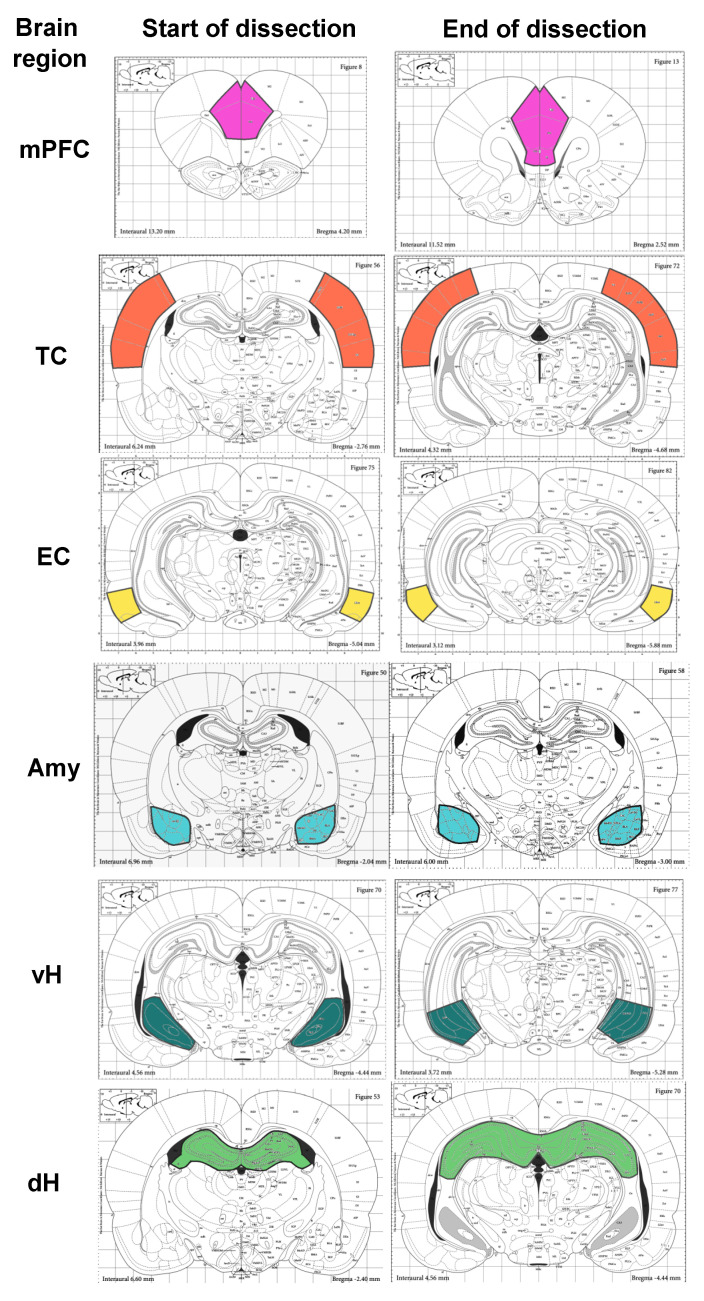
The scheme of brain dissection for RT-qPCR experiments. mPFC—medial prefrontal cortex; TC—temporal cortex; EC—entorhinal cortex; Amy—amygdala; dH, vH—dorsal and ventral areas of the hippocampus, respectively.

Brain samples were collected, quickly frozen, and stored at −70 °C. Brains were then put into the freezing microtome Thermo-scientific™ Microm HM525 (ThermoFisher Scientific, Berlin, Germany) for 1 h at −20 °C, and trimmed in the coronal direction with regions of interest captured by microspatule. The medial prefrontal (infralimbic, prelimbic, and anterior cingulate), entorhinal, and temporal cortical areas; the amygdala; and the dorsal and ventral regions of the hippocampus were dissected according to the scheme based on the rat brain atlas [28] (Figure 2).

Immediately after dissection, samples were homogenized in an appropriate volume of ExtractRNA reagent (Evrogen, Moscow, Russia) followed by single-step acid guanidiniumthiocyanate-phenol-chloroform extraction of total RNA [29] according to the manufacturer’s instruction. The RNA concentration (via 260 nm absorption) and purity (260/280 nm absorption ratio) were measured spectrophotometrically using a Nanodrop 2000 instrument (ThermoFisher Scientific, Waltham, MA, USA). The cDNA was synthesized from 1 μg of total RNA with 0.5 μg oligodT-primers (DNA Synthesis Ltd., Moscow, Russia), 20 units of ribonuclease inhibitor RNAsine (Sileks, Moscow, Russia), and 100 units of M-MLV reverse transcriptase (Promega Corp., Madison, WI, USA) according to the manufacturer’s instructions. Next, 10 µL of the solution containing RNA and primers was incubated 10 min at 70 °C and then quickly cooled to 4 °C. Then, 10 µL of a mix containing revertase in a 2× RT buffer, dNTPs, and RNAsine was added (to final volume 20 µL), and the reaction was carried out for 2 h at 42 °C. This was followed by 10 min at 65 °C for enzyme inactivation. The cDNA was diluted 10-fold before the PCR step.

### 2.3. qRT-PCR

This study evaluated the stability of nine housekeeping genes that are frequently used as references for gene expression normalization in the rat brain [9] and are responsible for different functions in the cell. The descriptions of genes tested and primer/probes used are in Table A1 in Appendix A. Three triplex qPCR assays validated in our previous work [30] were used: *Actb* + *Gapdh* + *B2m*; *Rpl13a* + *Sdha* + *Ppia*; and *Hprt1* + *Pgk1* + *Ywhaz*.

Multiplex qPCR reactions were optimized and fully described previously [30]. The reaction mix contained 0.8 µL of the cDNA sample, 0.75 units of TaqM-polymerase (Alkor-bio, Saint-Petersburg, Russia), 200 nM of specific forward and reverse primers, 100 nM (200 nM for *Actb*) TaqMan probes, 3.5 mM MgCl_2_, and 250 µM dATP/dTTP/dCTP/dGTP in 10 µL total volume of 1× TaqM-reaction buffer. Oligonucleotides were provided by DNA Synthesis Ltd. (Moscow, Russia). All reactions were duplicated and carried out with no reverse transcription and no template control samples on a C1000 Touch Thermal Cycler combined with the CFX96 real-time detection system (Bio-Rad, Hercules, CA, USA). Cycling conditions were set as follows: (1) hot start step at 95 °C for 15 min (recommended by enzyme manufacturer), (2) 5 cycles with 95 °C denaturation step for 5 s and 60 °C annealing/elongation step for 10 s (without plate read), (3) 35 cycles with 95 °C denaturation step for 5 s and 60 °C annealing/elongation step for 10 s, and (4) fluorescence plate read (about 13 s in the used instrument).

### 2.4. Data Analysis

The PCR curves were analyzed using the CFX Manager software (Bio-Rad Laboratories, Inc., Hercules, CA, USA). Quantification cycles (Cq) were determined by a single threshold. Raw means Cq data were imported to the RefFinder online tool (https://www.heartcure.com.au/reffinder/) to evaluate the reference gene stability. RefFinder utilizes four commonly used algorithms for reference gene validation (geNorm, NormFinder, BestKeeper, and the comparative Delta-Ct method). It creates a ranking based on geometric averaging of the ranks obtained by the four algorithms [31].

GeNorm is based on the principle that the expression ratio of two appropriate reference genes should be unaffected by the experimental conditions. The geNorm calculates a stability value (M) by averaging pair-wise variations for each reference gene, and then comparing this to other control genes with stepwise exclusion of the least-stable gene and M recalculation. The lowest M-value indicates the most stable pair of genes within a tested panel and experiment condition, whereas the highest M-value indicates the least-stable gene [10].

The comparative Delta-Ct method contrasts the relative expression of pairs of genes within each sample. The stability of the candidate reference gene is ranked according to the repeatability of the gene expression difference; i.e., the gene with the lowest mean deltaCT standard deviation is considered to be the most stable [13].

BestKeeper uses pair-wise comparisons of raw Cq values for each gene to define the most stable reference gene [12]. The BestKeeper tool generates an index using the geometric mean of each candidate gene’s Cp value. This index can be used to rank the best reference genes, since stable reference gene expression correlates with the BestKeeper index. BestKeeper also calculates the coefficient of variation (CV), the standard deviation (SD) of the Cq values, and the coefficient of correlation (R). Stable reference genes show high R-values and low CV and SD.

NormFinder is based on a mathematical model of gene expression to determine the most stable reference gene and the best combination of two reference genes. NormFinder software calculates a stability value of the candidate reference genes based on an estimation of the overall variation of the candidate reference genes and the variation between subgroups of the sample set. The algorithm has been shown to be less sensitive to coregulation. Therefore, the algorithm does not require knowledge of whether genes are responsible for different functions in the cell [11].

## 3. Results

We evaluated the expression stability of nine housekeeping genes in the rat brain after PTZ-induced tonic-clonic seizures at P20–22.

**Figure 3:**
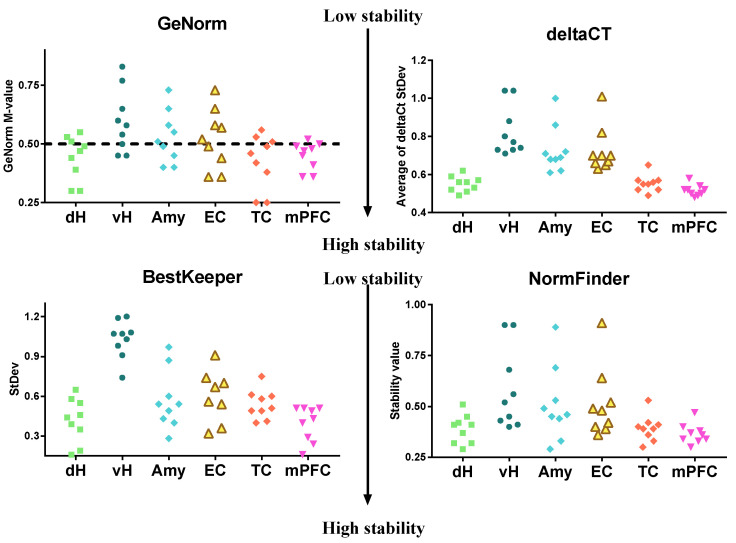
Reference genes’ stability values obtained for nine housekeeping genes in different regions of the brain during one week after PTZ-induced seizures. Gene expression stability in overall samples of PTZ-treated (70 mg/kg i.p., P21) and vehicle control animals at different time points (3 h, 1, 3, 7 days; n: 4–7 per group) was assessed by RefFinder online tool. The calculation was performed for overall samples (control and experimental, four time points) within one brain region. The dashed line shows the cut-off value (M > 0.5) for the GeNorm algorithm; values above the dashed line indicate unstably expressed genes. dH and vH—dorsal and ventral areas of the hippocampus, respectively; Amy—Amygdala; EC, TC, mPFC—entorhinal, temporal, and medial prefrontal cortical areas respectively.

Gene expression was analyzed in PTZ-treated and control animals at different time points (3 h and 1, 3, and 7 days) in various brain regions. Figure 3 summarizes the stability values obtained by four algorithms assessing six brain areas in control and experimental animals at all time points. We found that tested reference genes demonstrated high stability in the medial prefrontal and temporal cortex and the dorsal hippocampus. Low stability was observed in the ventral hippocampus, amygdala, and entorhinal cortex.

**Figure 4:**
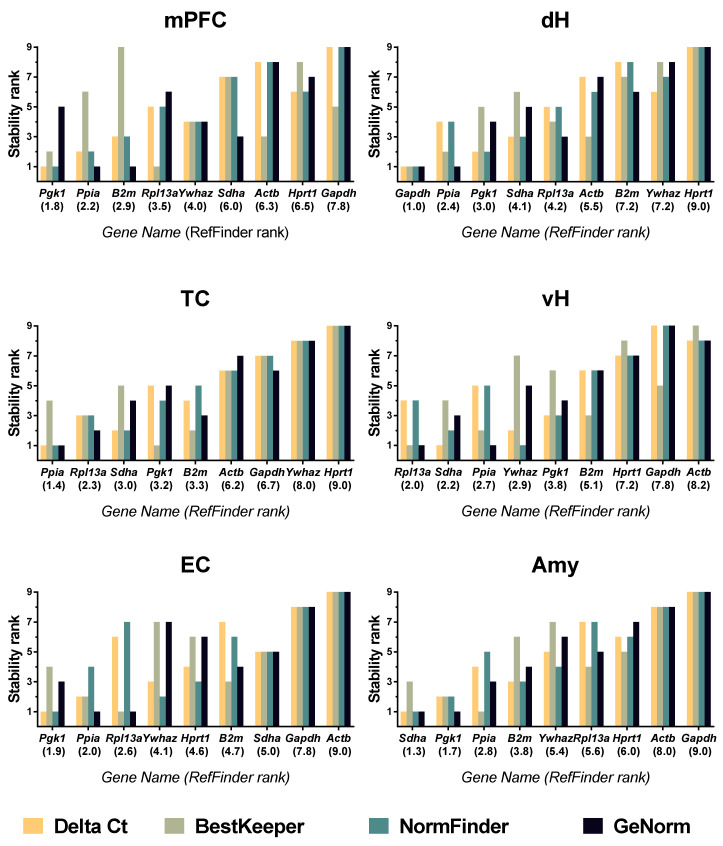
The reference gene stability rankings in different regions of the brain during one week after PTZ-induced seizures in juvenile rats. Gene expression stability in PTZ-treated (70 mg/kg i.p., P21) and vehicle control animals at different time points (3 h, 1, 3, 7 days; n: 4–7 per group) was assessed by RefFinder online tool. mPFC—medial prefrontal cortex; TC—temporal cortex; EC—entorhinal cortex; Amy—amygdala; dH, vH—dorsal and ventral areas of the hippocampus, respectively.

A comprehensive ranking provided by the RefFinder tool based on the four algorithms indicates that, in the week after PTZ-induced seizures in juvenile rats, *Pgk1*, *Ppia*, and *B2m* are the most stably expressed within the medial prefrontal cortex, and *Ppia*, *Rpl13a*, and *Sdha* are the most stably expressed within the temporal cortex (Figure 4).

In the entorhinal cortex, as in the medial prefrontal cortex, RefFinder identified *Pgk1* and *Ppia* to be the most valid reference genes from the tested panel in immature rats after PTZ-induced seizures. The third most stably expressed gene was *Rpl13a*, which also demonstrated high stability within the temporal cortex. *Sdha*, *Pgk1*, and *Ppia* were identified as the most stably expressed within the amygdalae of control and experimental rats. RefFinder identified *Gapdh*, *Ppia*, and *Pgk1* as the most stably expressed reference genes within the dorsal hippocampus. In the ventral hippocampus, *Rpl13a*, *Sdha,* and *Ppia* were found to be the most suitable reference genes.

In the week following PTZ-induced seizures, based on geNorm analysis, we found unstable genes within the analyzed brain regions, i.e., genes with geNorm M-values exceeding the 0.5 cut-off level [10] (Supplementary Materials Figures S1–S6). *Gapdh* demonstrated an inappropriate geNorm M-value in the medial prefrontal cortex. The expressions of *Actb*, *Ywhaz*, and *Hprt1* were unstable in the temporal cortex. In the entorhinal cortex, five of nine tested genes were found to be unsuitable for RT-qPCR analysis: *Sdha*, *Hprt1*, *Ywhaz*, *Gapdh*, and *Actb*. Five genes (*Rpl13a*, *Ywhaz*, *Hprt1*, *Gapdh*, and *Actb*) demonstrated inappropriately high expression variability in the amygdala. *Actb*, *Ywhaz*, and *Hprt1* showed unstable expression in the dorsal hippocampus. The expressions of six genes (*Pgk1*, *Ywhaz*, *B2m*, *Hprt1*, *Actb*, and *Gapdh*) were unsuitable for RT-qPCR analysis in the ventral hippocampus.

We have also analyzed reference gene expression stability separately for different time points after acute seizures (Supplementary Materials Figures S7–S12). Rankings differed slightly depending on analyzed time points; however, the least-stable genes were mostly the same throughout all experiments.

## 4. Discussion

In our study, we evaluated reference gene stability within different cortical and subcortical brain regions of immature rats using the PTZ model of acute seizures. Our data reveal that reference gene stability rankings vary across brain regions, including in different areas of the neocortex and the dorsal vs. ventral hippocampus. *Ppia* was found to be one of the most stable genes in all six analyzed brain regions, *Pgk1* in four regions, and *Rpl13a* and *Sdha* in three of six regions. Based on the geNorm 0.5 cut-off M-value, *Hprt1*, *Actb*, and *Ywhaz* were unsuitable for RT-qPCR in five of the six analyzed brain areas, and *Gapdh* was unsuitable in four areas.

We could not find in PubMed any previous study analyzing gene stability in the brain using the PTZ model. Only a few works investigated the stability of housekeeping gene expression using other animal models of seizure or epilepsy: after neonatal (P10) febrile seizures [2]; after short (30 min) and long (8 h) perforant pathway stimulation [15]; after systemic/intrahippocampal pilocarpine injection [32]; and in the kainic acid model of temporal lobe epilepsy [7,15]. These works analyzed the entire hippocampus [15,32] or dental gyrus of the hippocampus [2]; an exception was the study by Crans et al. (2019) [7], where expression stability was analyzed in the hippocampal tissue and in the neocortex. However, the exact areas of the cortex analyzed were not specified in the article.

Seizures differentially affect different brain regions [21,27,33,34]. Loss of neurons and gliosis occurs in specific brain areas depending on the model used [35,36,37,38]. We also cannot exclude the possibility that expression stability may be affected by developmental factors, and our results are specific for young rats. Thus, direct comparison of results of previous studies on reference gene expression stability in rat experimental seizure models to our data should be done with caution due to the differences in the examined brain regions, seizure models, and ages of the animals.

*Gapdh* and *Actb*, which are widely used as reference genes [9], are unstable in most brain areas in the PTZ seizure model, according to our data. These genes also demonstrate unstable expression in various experimental conditions, including febrile seizures, the valproic acid model of autism, performant pathway stimulation seizures, and the latent phase of the kainate epilepsy model [2,3,9,15,39]. Therefore, *Gapdh* and *Actb* should be avoided for mRNA expression normalization as single reference genes in seizure models.

The most stably expressed genes across the brain regions tested in our study were *Ppia* and *Pgk1.* The hippocampal mRNA expressions of *Ppia* and *Pgk1* were also reported to be stable in the asphyxial cardiac arrest model and the latent phase of the kainate epilepsy model [7,15]. *Ppia* expression was stable in the dentate gyrus in the febrile seizure model [2]. However, *Ppia* expression was unstable after seizures induced by performant pathway stimulation [15], and in the pilocarpine model of temporal lobe epilepsy [32]. Interestingly, two glycolysis related genes, *Gapdh* and *Pgk1,* demonstrate different expression stability in most of the examined brain areas (Figure 4). Similarly to our results, significant variation between *Gapdh* and *Pgk1* expression stabilities was found in the tissue culture of rat intervertebral disk [40]. It is worth noting that both enzymes have multiple non-canonical functions apart from glycolysis. PGK1 is known to act as a proteinkinase [41]; GAPDH participates in apoptosis, DNA repair, transcriptional regulation, and nuclear membrane assembly [42]. Upregulated GAPDH protein expression was reported in the mouse hippocampus after kainite-induced seizures [43]. Since *Gapdh* and *Pgk1* gene products differ by their multiple non-canonical functions, the difference in the expression regulatory pathway can be suggested.

Here, we report that not only rankings but overall expression stability of the tested gene panel are firmly brain-region-dependent. In our model, we could see that housekeeping gene expression was stable in the medial prefrontal cortex, temporal cortex, and dorsal hippocampus. In contrast, in the ventral hippocampus, entorhinal cortex, and amygdala, most of the tested genes demonstrated low stability (Figure 4). This may reflect a different degree of involvement for each brain region in the pathological processes occurring after PTZ-induced seizures. Certain fMRI and c-Fos immunohistochemical studies have revealed that a wide spectrum of brain areas are activated during PTZ-induced acute seizures, including different areas of the neocortex, hippocampus, amygdala, and other subcortical regions [25,26]. However, the extents of impairment in distinct brain regions and the roles of said impairments in cognitive deficit development after PTZ-induced seizures remain unclear.

Our data suggest that the amygdala, entorhinal cortex, and ventral hippocampus, which are located near each other, are strongly affected by PTZ-induced tonic-clonic seizures. The entorhinal cortex is interconnected with the hippocampus, and the ventral area of the hippocampus and amygdala are also largely interconnected [44]. These areas are believed to be involved in seizure generation and propagation. The hippocampus, amygdala, and entorhinal cortex are highly susceptible to seizurogenic insults. In many seizure models, these brain structures generate seizure activity [45,46].

It should be noted that the dorsal and ventral areas of the hippocampus significantly differ in functional properties and gene expression profiles [44]. Ventral hippocampal cells are more susceptible to generating epileptiform activity than neurons in the dorsal hippocampus [47]. The unstable housekeeping gene expression observed in our experiments may also be explained by stress-related plasticity. The ventral hippocampus is known to mediate the stress response and be more susceptible to stress-induced neuroinflammation than the dorsal hippocampus [48]. Together with existing data about significant differences in transcriptome, connectivity, and realized functions between the dorsal and ventral hippocampus [44], our data indicate the need for differential reference gene selection for the two hippocampal areas.

We provided validation of suitable reference genes for mRNA expression analysis in different cortical and subcortical brain regions of immature rats during the week after PTZ-induced seizures. We found that housekeeping gene expression had less stability in the ventral hippocampus, amygdala, and entorhinal cortex, indicating their high susceptibility to the detrimental effects of seizures in the PTZ model. The data provided on reference gene stability would be useful for future experiments evaluating short and long-term effects of PTZ-induced seizures on gene expression across the brains of immature rats. Our data obtained with the model of juvenile seizures indicate the need for a differential selection of reference genes across brain regions, including both the dorsal and ventral hippocampus.

## Supplementary Materials

The following are available online at https://www.mdpi.com/2227-9059/8/8/239/s1. Figure S1: The reference gene stability within the medial prefrontal cortexes of control and exposed to PTZ-induced seizures juvenile rats. Figure S2: The reference gene stability within the temporal cortexes of control and exposed to PTZ-induced seizures juvenile rats. Figure S3: The reference gene stability within the entorhinal cortexes of control and exposed to PTZ-induced seizures juvenile rats. Figure S4: The reference gene stability within the amygdalae of control and exposed to PTZ-induced seizures juvenile rats. Figure S5: The reference gene stability within the dorsal hippocampi of control and exposed to PTZ-induced seizures juvenile rats. Figure S6: The reference gene stability within the ventral hippocampi of control and exposed to PTZ-induced seizures juvenile rats. Figure S7: The reference gene stability within the mPFCs of control and PTZ-treated rats at different time points after seizures. Figure S8: The reference gene stability within the temporal cortexes of control and PTZ-treated rats at different time points after seizures. Figure S9: The reference gene stability within the entorhinal cortexes of control and PTZ-treated rats at different time points after seizures. Figure S10: The reference gene stability within the amygdalae of control and PTZ-treated rats at different time points after seizures. Figure S11: The reference gene stability within the dorsal hippocampi of control and PTZ-treated rats at different time points after seizures. Figure S12: The reference gene stability within the ventral hippocampi of control and PTZ-treated rats at different time points after seizures.

## Appendix A

**Table A1:** Primers and probes used for RT-PCR.

Gene Symbol RefSeq Accession Number	The Encoded Protein and Its Function	Primer 5′–3′ (Forward, Reverse, Probe)	Amplicon Length and Position on mRNA	Reference
*Gapdh*^1^ NM_017008	Glyceraldehyde-3-phosphate dehydrogenase Glycolytic enzyme	TGCACCACCAACTGCTTAG GGATGCAGGGATGATGTTC ATCACGCCACAGCTTTCCAGAGGG *	177 bp (523–699)	[49]
*B2m*^1^ NM_012512	Beta-2-microglobulin Assembly and surface expression of MHC class I molecules	TGCCATTCAGAAAACTCCCC GAGGAAGTTGGGCTTCCCATT ATTCAAGTGTACTCTCGCCATCCACCG&	73 bp (77–149)	[50]
*Actb*^1^ NM_031144	Beta-actin Cytoskeletal protein	TGTCACCAACTGGGACGATA GGGGTGTTGAAGGTCTCAAA CGTGTGGCCCCTGAGGAGCAC #	165 bp (303–467)	[51] (forward and reverse primers) [30] (hydrolysis probe)
*Ppia*^2^ NM_017101	Peptidyl prolyl isomerase A, cyclophilin A Protein folding	AGGATTCATGTGCCAGGGTG CTCAGTCTTGGCAGTGCAGA CACGCCATAATGGCACTGGTGGCA &	187 bp (216–402)	[33]
*Rpl13a*^2^ NM_173340	Ribosomal protein L13A 60S ribosomal subunit protein	GGATCCCTCCACCCTATGACA CTGGTACTTCCACCCGACCTC CTGCCCTCAAGGTTGTGCGGCT #	131 bp (334–464)	[2] (forward and reverse primers) [30] (hydrolysis probe)
*Sdha*^3^ NM_130428	Succinate dehydrogenase complex, subunit a, flavoprotein Component of electron transport chain	AGACGTTTGACAGGGGAATG TCATCAATCCGCACCTTGTA ACCTGGTGGAGACGCTGGAGCT *	160 bp (1666–1825)	[52] (forward and reverse primers) [30] (hydrolysis probe)
*Ywhaz*^3^ NM_013011	Tyrosine 3-monooxygenase/tryptophan 5-monooxygenase activation protein, zeta polypeptide Cell signaling	GATGAAGCCATTGCTGAACTTG GTCTCCTTGGGTATCCGATGTC TGAAGAGTCGTACAAAGACAGCACGC &	117 bp (650–766)	[14] (forward and reverse primers) [30] (hydrolysis probe)
*Pgk1*^3^ NM_053291	Phosphoglycerate kinase 1 Glycolytic enzyme	ATGCAAAGACTGGCCAAGCTAC AGCCACAGCCTCAGCATATTTC TGCTGGCTGGATGGGCTTGGA *	104 bp (888–991)	[14] (forward and reverse primers) [30] (hydrolysis probe)
*Hprt*^3^ NM_012583	Hypoxanthine phosphoribosyl transferase 1 Purine synthesis	TCCTCAGACCGCTTTTCCCGC TCATCATCACTAATCACGACGCTGG CCGACCGGTTCTGTCATGTCGACCCT #	80 bp (28–107)	[53] (forward and reverse primers) [30] (hydrolysis probe)

* Labeled with R6G fluorescent dye (5′) and BHQ2 quencher (3′). # Labeled with FAM fluorescent dye (5′) and BHQ1 quencher. & Labeled with Rox fluorescent dye (5′) and BHQ1 quencher (3′). ^1, 2, 3^ —assays with the same indeces were run in a single multiplex reaction.
