# Supplementary material for: Reference Gene Validation in the Brain Regions of Young Rats after Pentylenetetrazole-Induced Seizures
Source: Biomedicines. 2020 Jul 23;8(8):239. doi: 10.3390/biomedicines8080239 (PMC7460155; doi:10.3390/biomedicines8080239)
Supplement: Supplementary file 1 [file biomedicines-08-00239-s001.zip › Figures S1-S6_R1.pdf]

Medial Prefrontal Cortex

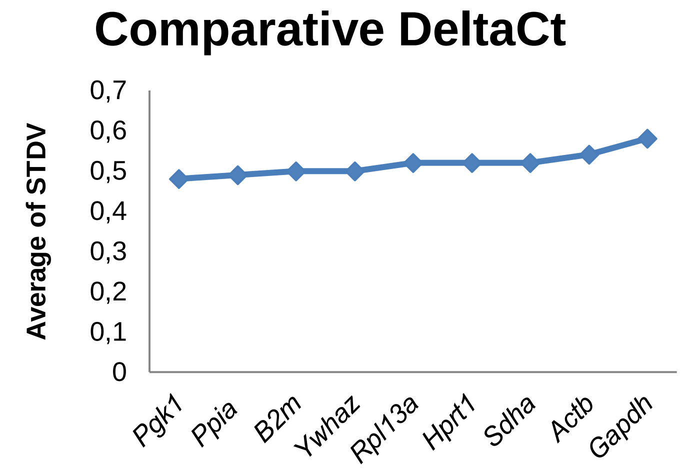

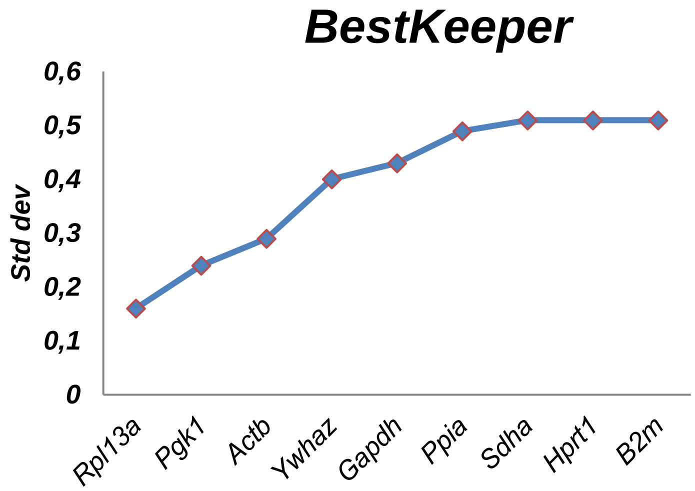

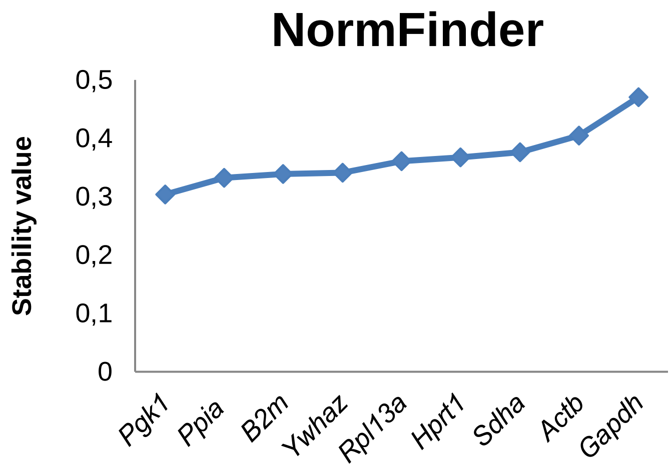

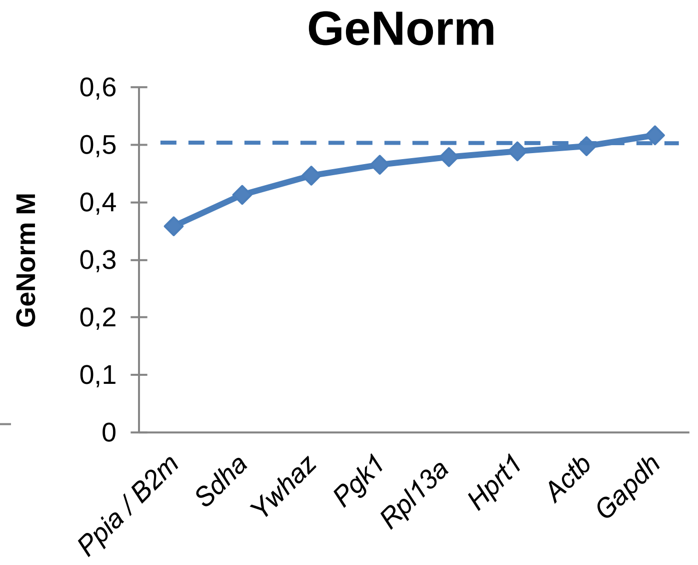

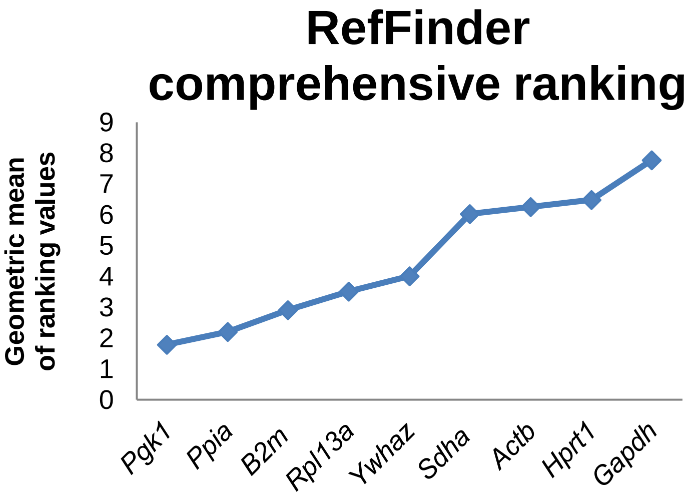

Fig. S1. The reference gene stability within the medial prefrontal cortex of control and exposed to PTZ-induced seizures juvenile rats. Seizures was induced at P21. Gene expression stability in PTZ-treated and vehicle control animals was assessed by RefFinder online tool.

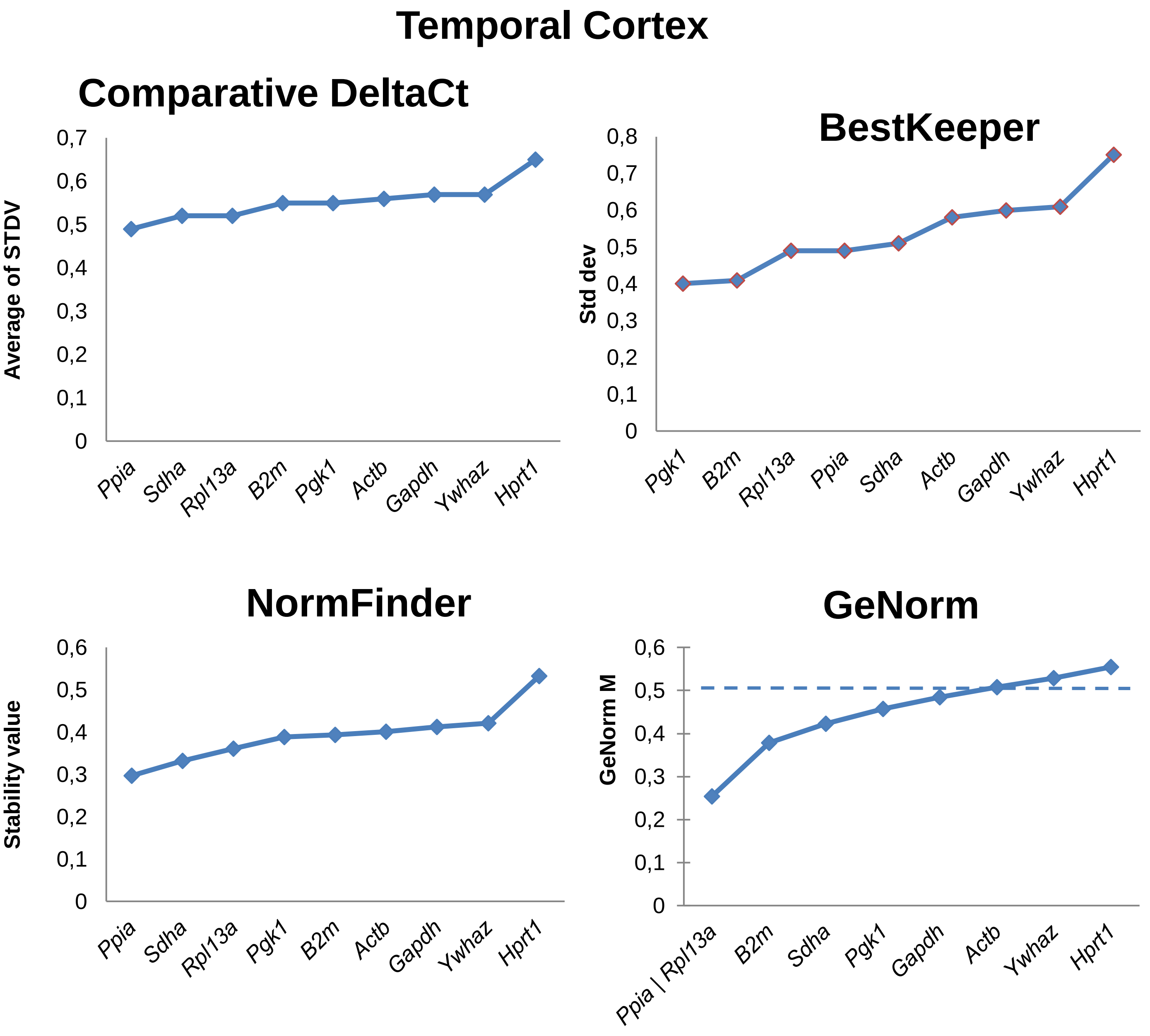

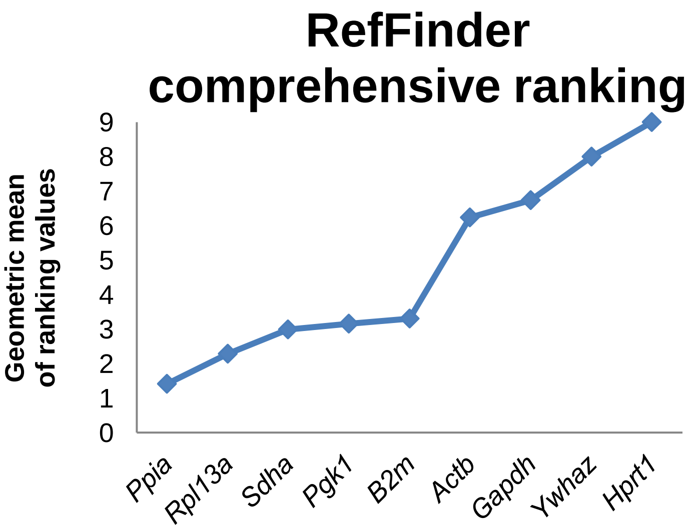

Fig. S2. The reference gene stability within the temporal cortex of control and exposed to PTZ-induced seizures juvenile rats. Seizures was induced at P21. Gene expression stability in PTZ-treated and vehicle control animals was assessed by RefFinder online tool.

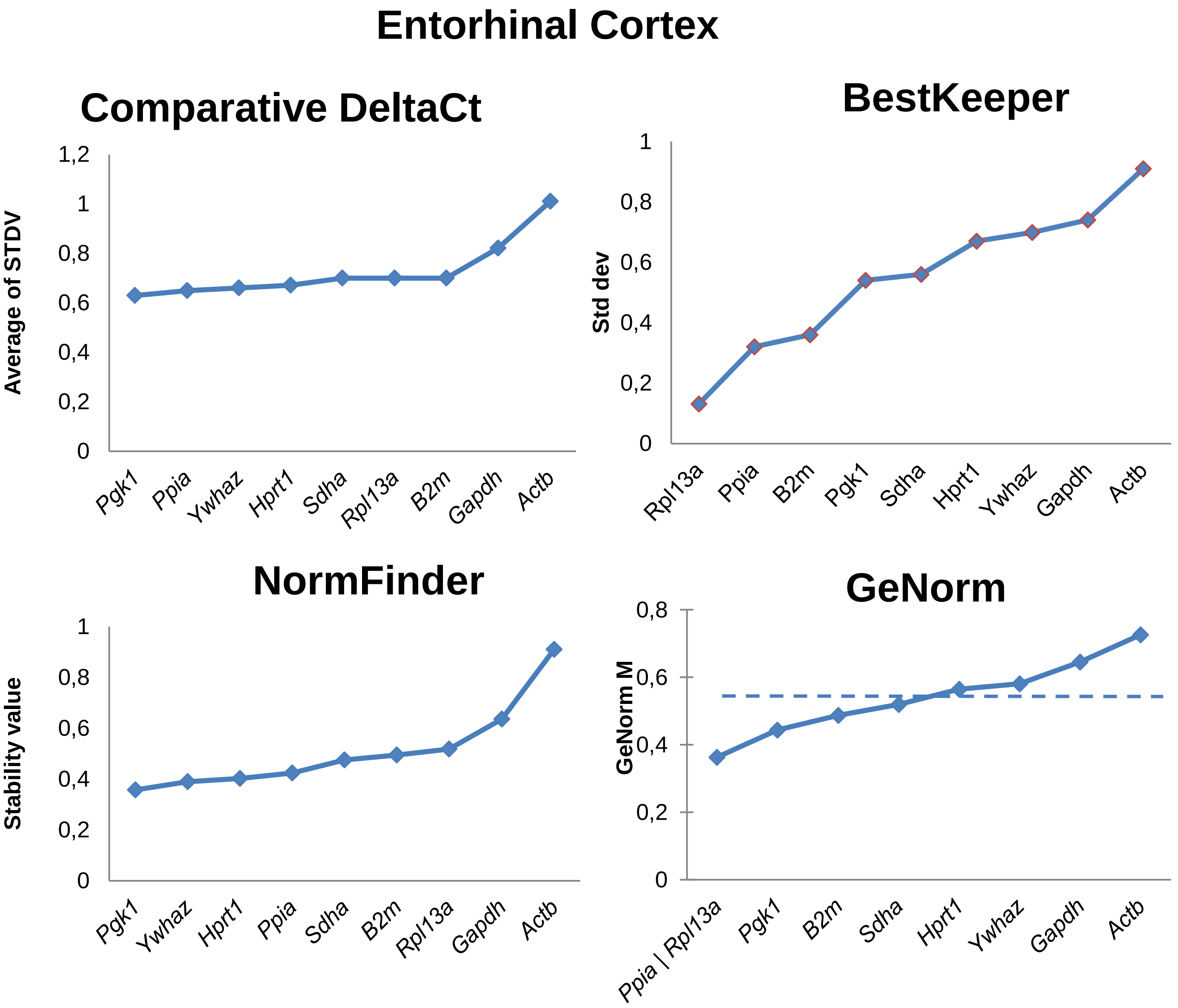

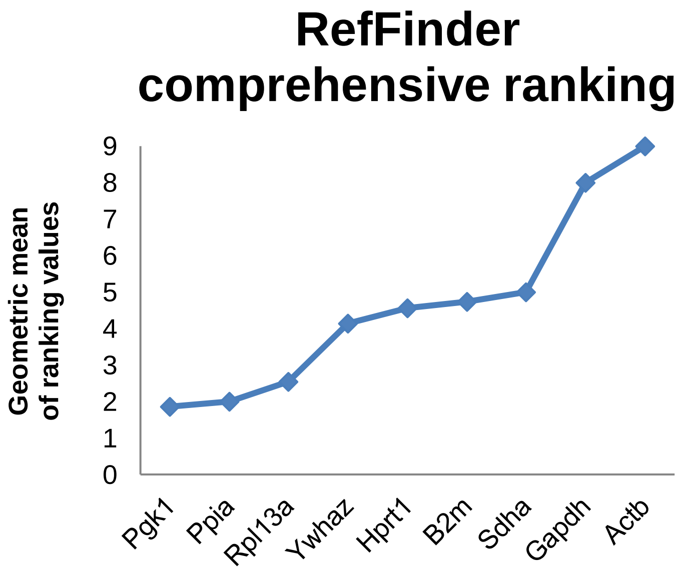

Fig. S3. The reference gene stability within the entorhinal cortex of control and exposed to PTZ-induced seizures juvenile rats. Seizures was induced at P21. Gene expression stability in PTZ-treated and vehicle control animals was assessed by RefFinder online tool.

Amygdala

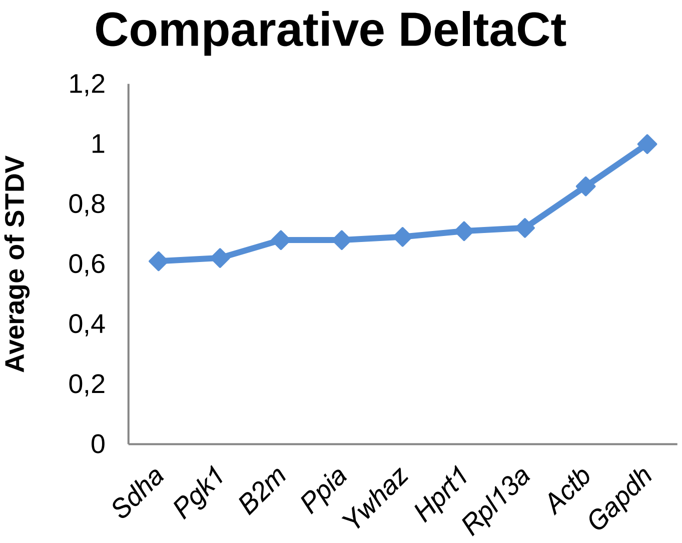

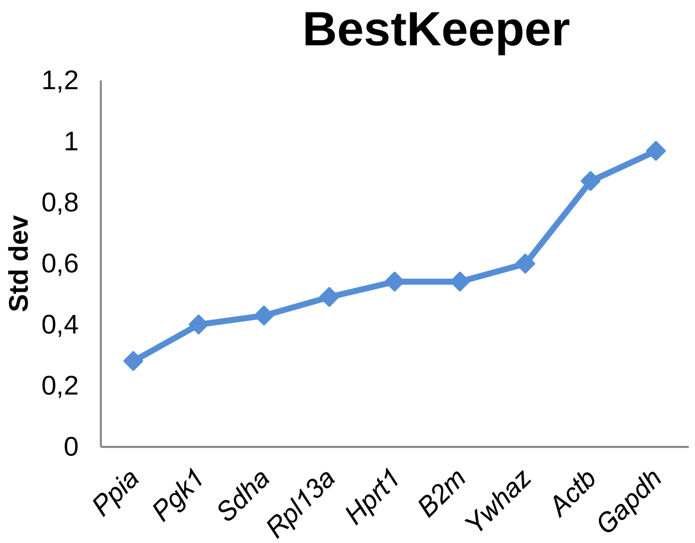

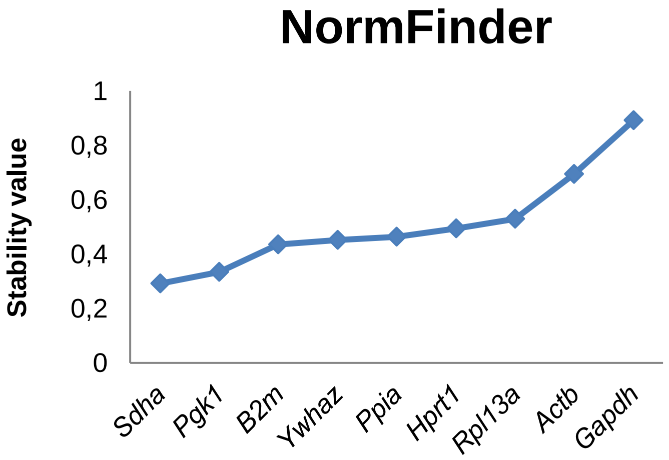

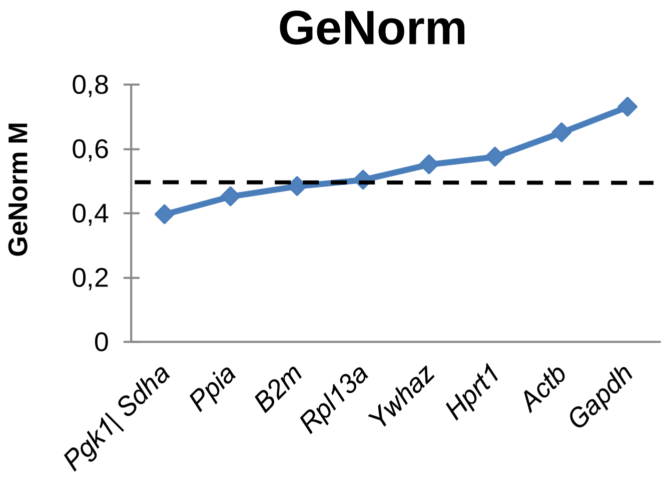

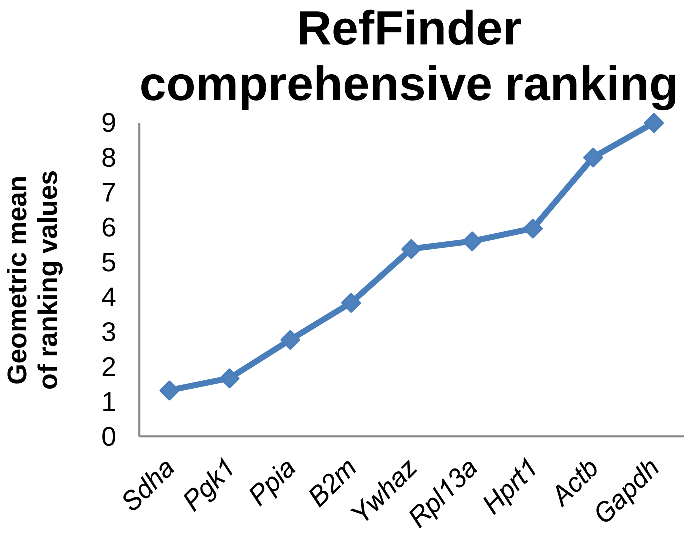

Fig. S4. The reference gene stability within the amygdala of control and exposed to PTZ-induced seizures juvenile rats. Seizures was induced at P21. Gene expression stability in PTZ-treated and vehicle control animals was assessed by RefFinder online tool.

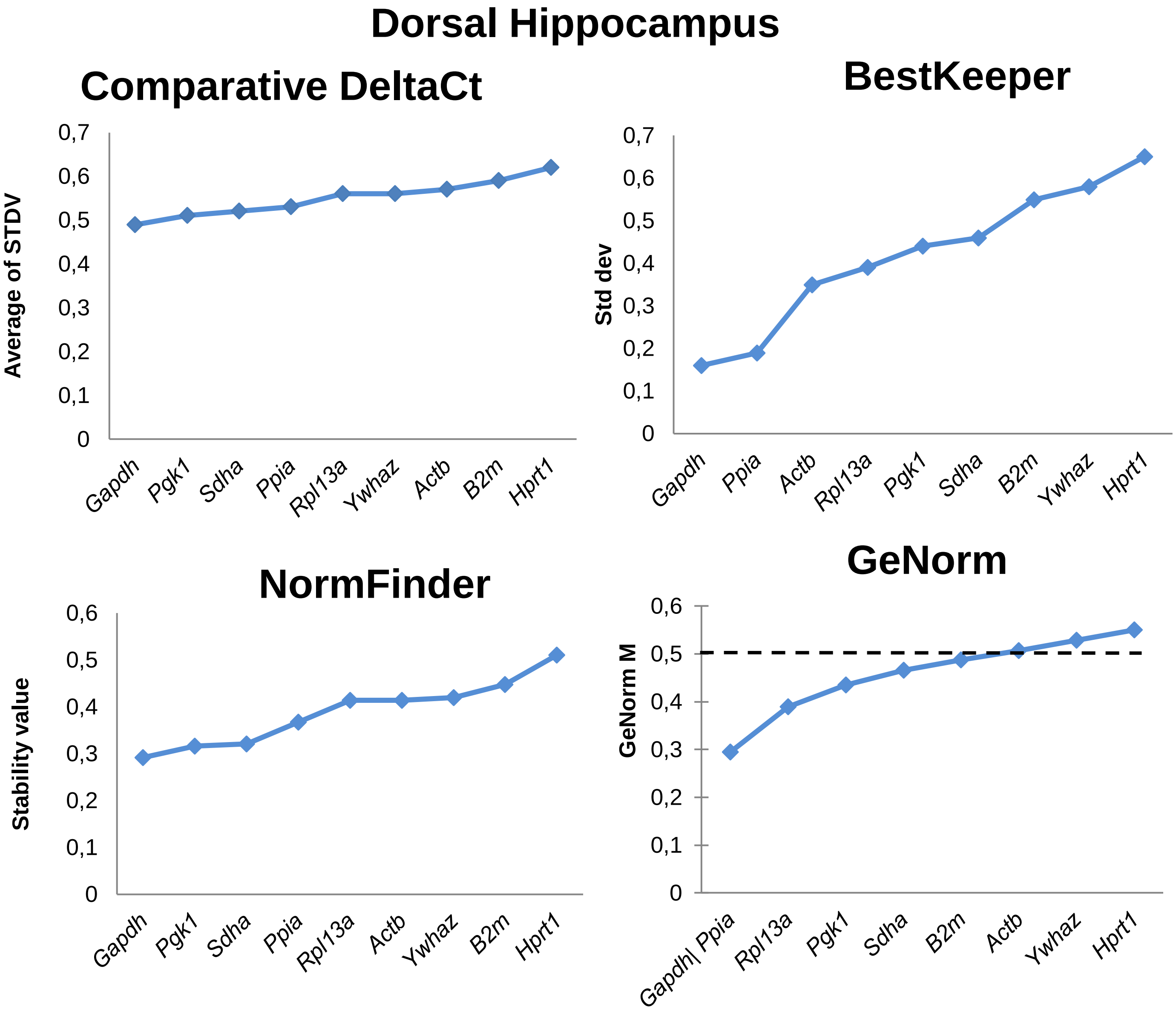

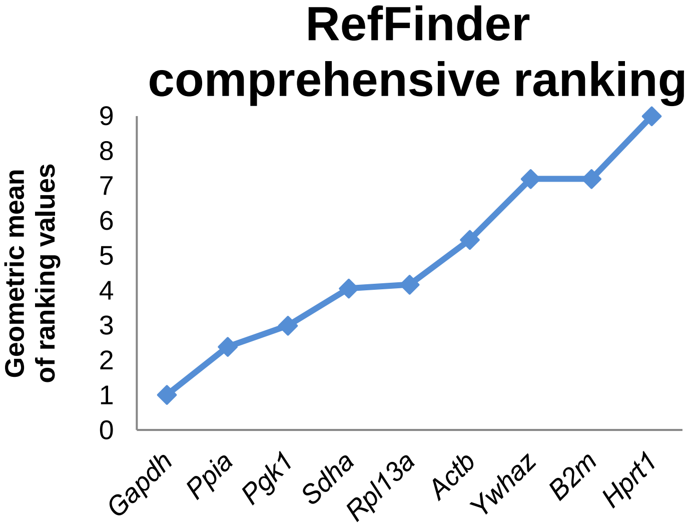

Fig. S5. The reference gene stability within the dorsal hippocampus of control and exposed to PTZ-induced seizures juvenile rats. Seizures was induced at P21. Gene expression stability in PTZ-treated and vehicle control animals was assessed by RefFinder online tool.

Ventral Hippocampus

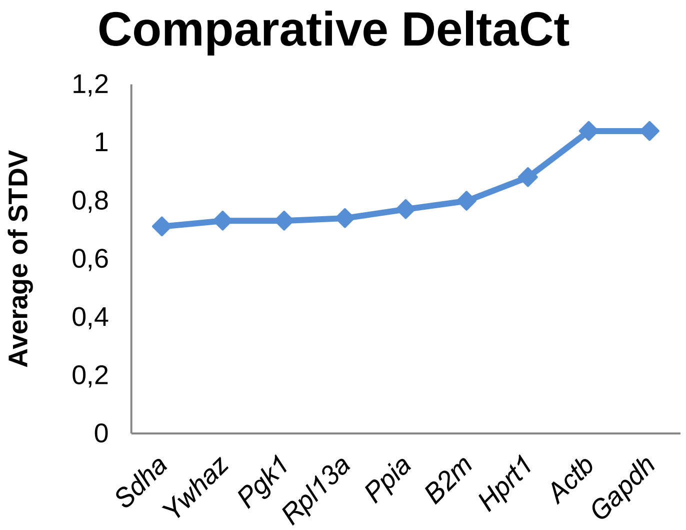

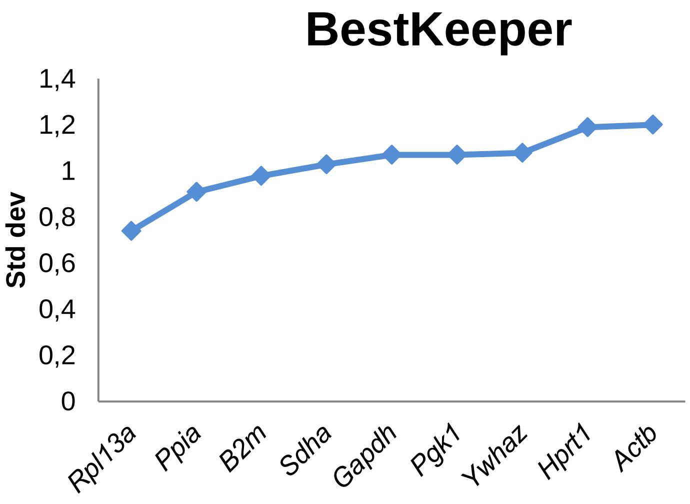

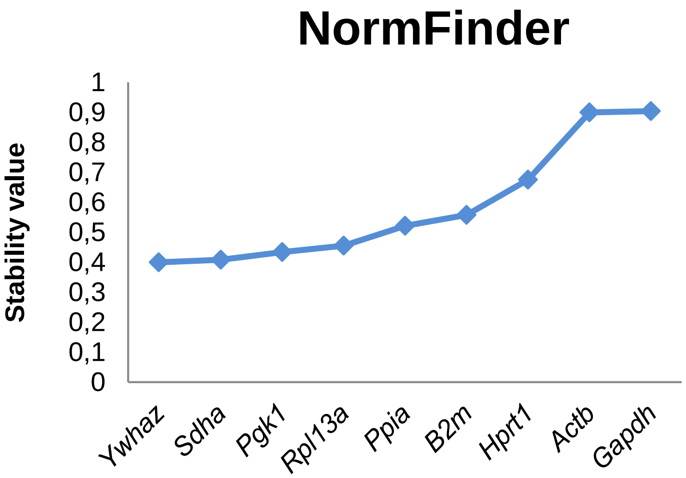

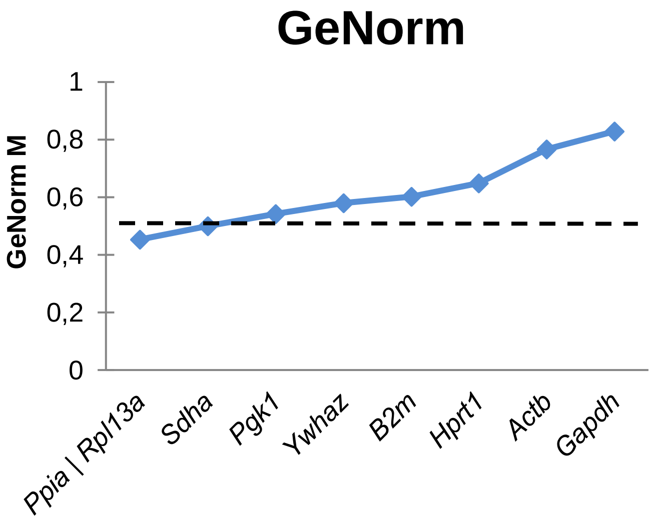

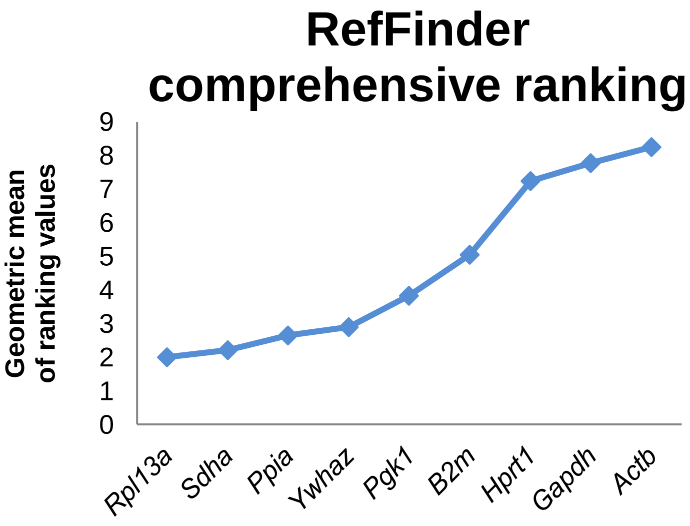

Fig. S6. The reference gene stability within the ventral hippocampus of control and exposed to PTZ-induced seizures juvenile rats. Seizures was induced at P21. Gene expression stability in PTZ-treated and vehicle control animals was assessed by RefFinder online tool.
