# Supplementary material for: Reference Gene Validation in the Brain Regions of Young Rats after Pentylenetetrazole-Induced Seizures
Source: Biomedicines. 2020 Jul 23;8(8):239. doi: 10.3390/biomedicines8080239 (PMC7460155; doi:10.3390/biomedicines8080239)
Supplement: Supplementary file 1 [file biomedicines-08-00239-s001.zip › Figures S7-S12_R1.pdf]

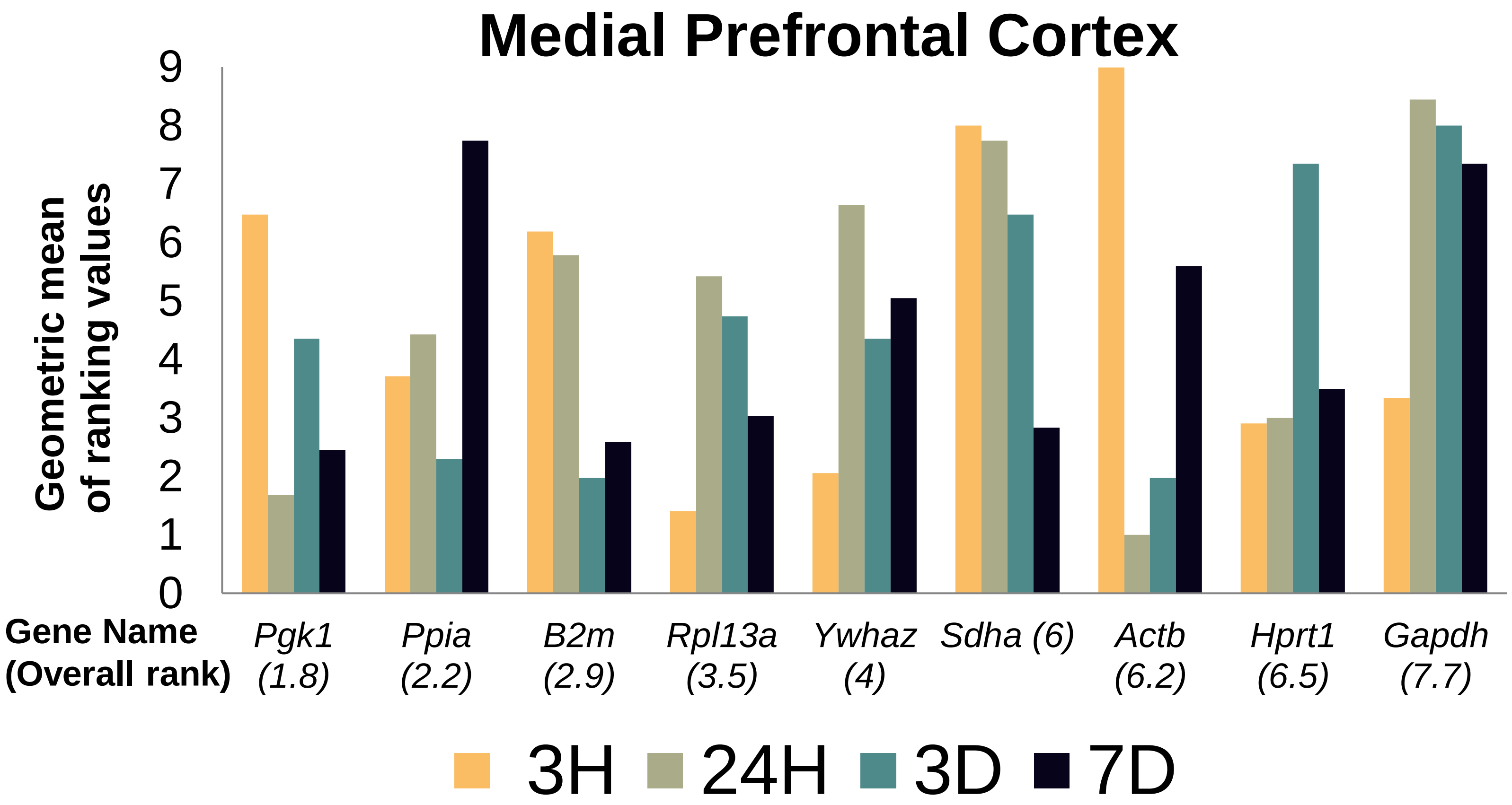

Time after Seizures	RefFinder Comprehensive ranking (Most stable→Least stable)								
**3 h**	*Rpl13a*	*Ywhaz*	*Hprt1*	*Gapdh*	*Ppia*	*B2m*	*Pgk1*	*Sdha*	*Actb*
**24 h**	*Actb*	*Pgk1*	*Hprt1*	*Ppia*	*Rpl13a*	*B2m*	*Ywhaz*	*Sdha*	*Gapdh*
**3 days**	*B2m*	*Actb*	*Ppia*	*Ywhaz*	*Pgk1*	*Rpl13a*	*Sdha*	*Hprt1*	*Gapdh*
**7 days**	*Pgk1*	*B2m*	*Sdha*	*Rpl13a*	*Hprt1*	*Ywhaz*	*Actb*	*Gapdh*	*Ppia*
**Overall**	***Pgk1***	***Ppia***	***B2m***	***Rpl13a***	***Ywhaz***	***Sdha***	***Actb***	***Hprt1***	***Gapdh***

Figure S7. The reference gene stability within the mPFC of control and PTZ-treated rats at different time points after seizures. Seizures was induced at P21. Gene expression stability in PTZ-treated and vehicle control animals at different time points (3 h, 1, 3, 7 days; n – 4-7 per group) was assessed by RefFinder online tool.

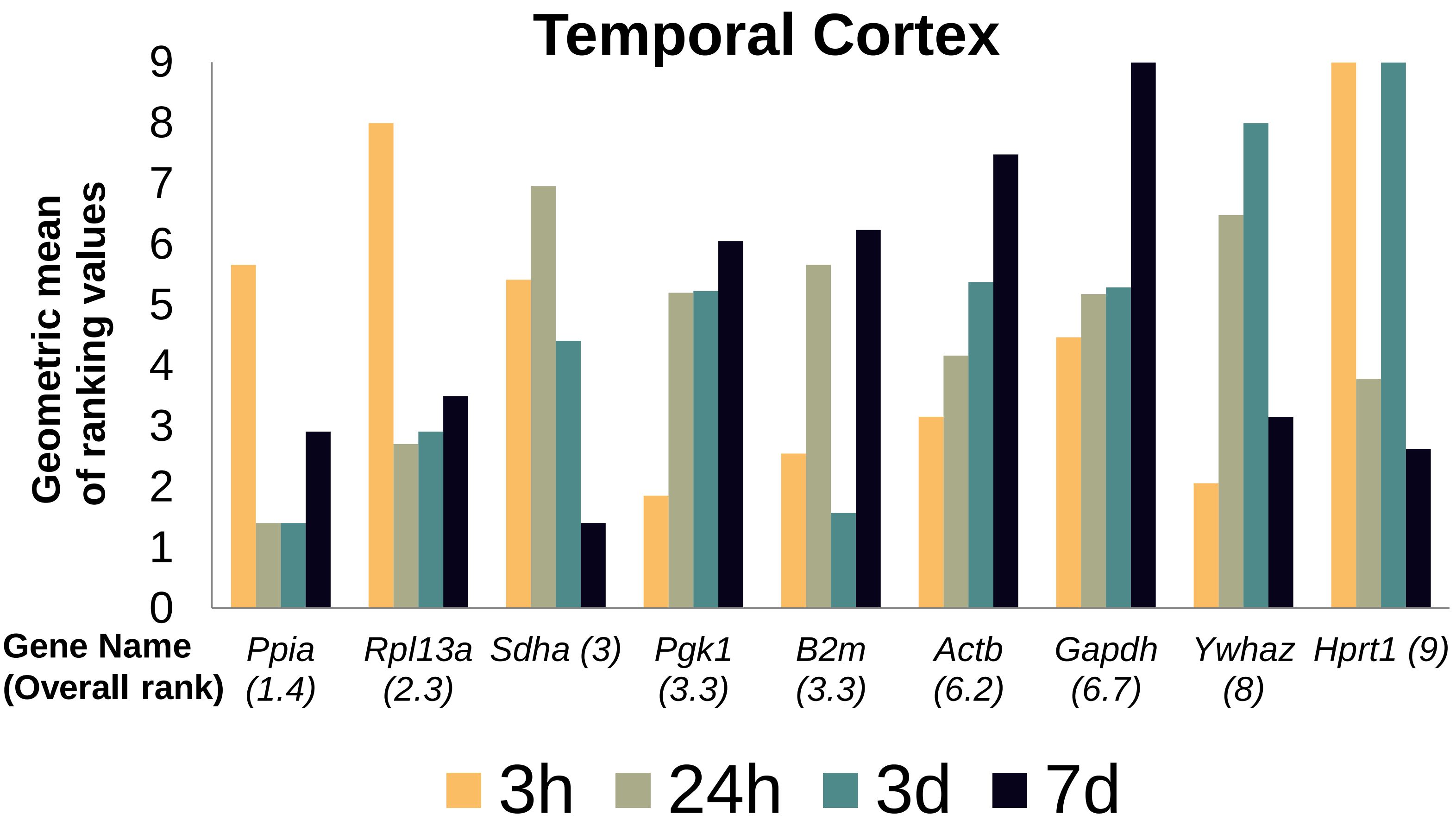

Time after Seizures	RefFinder Comprehensive ranking (Most stable→Least stable)								
**3 h**	*Pgk1*	*Ywhaz*	*B2m*	*Actb*	*Gapdh*	*Sdha*	*Ppia*	*Rpl13a*	*Hprt1*
**24 h**	*Ppia*	*Rpl13a*	*Hprt1*	*Actb*	*Gapdh*	*Pgk1*	*B2m*	*Ywhaz*	*Sdha*
**3 days**	*Ppia*	*B2m*	*Rpl13a*	*Sdha*	*Pgk1*	*Gapdh*	*Actb*	*Ywhaz*	*Hprt1*
**7 days**	*Sdha*	*Hprt1*	*Ppia*	*Ywhaz*	*Rpl13a*	*Pgk1*	*B2m*	*Actb*	*Gapdh*
**Overall**	***Ppia***	***Rpl13a***	***Sdha***	***Pgk1***	***B2m***	***Actb***	***Gapdh***	***Ywhaz***	***Hprt1***

Figure S8. The reference gene stability within the temporal cortex of control and PTZ-treated rats at different time points after seizures. Seizures was induced at P21. Gene expression stability in PTZ-treated and vehicle control animals at different time points (3 h, 1, 3, 7 days; n – 4-7 per group) was assessed by RefFinder online tool.

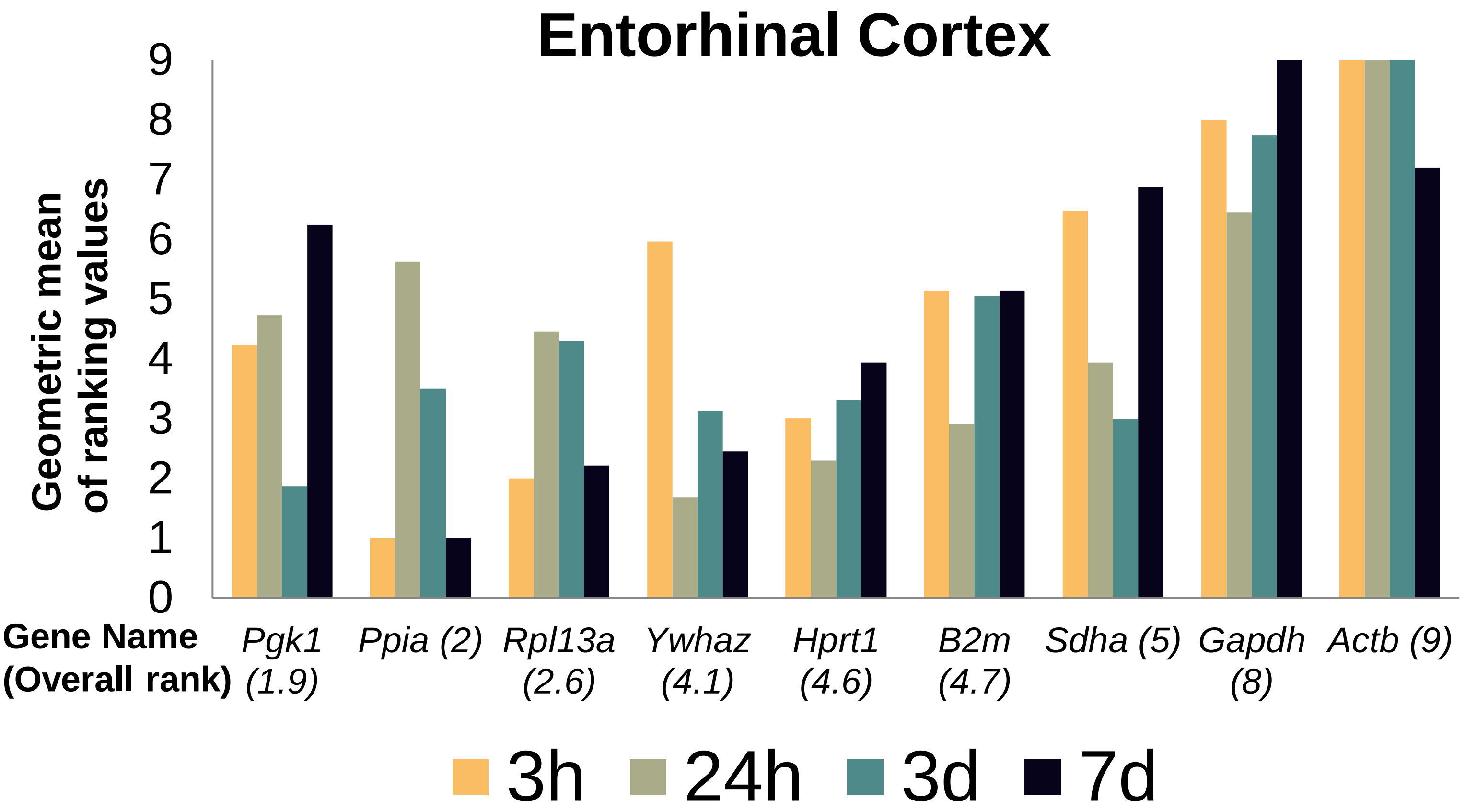

Time after Seizures	RefFinder Comprehensive ranking (Most stable→Least stable)								
**3 h**	*Ppia*	*Rpl13a*	*Hprt1*	*Pgk1*	*B2m*	*Ywhaz*	*Sdha*	*Gapdh*	*Actb*
**24 h**	*Ywhaz*	*Hprt1*	*B2m*	*Sdha*	*Rpl13a*	*Pgk1*	*Ppia*	*Gapdh*	*Actb*
**3 days**	*Pgk1*	*Sdha*	*Ywhaz*	*Hprt1*	*Ppia*	*Rpl13a*	*B2m*	*Gapdh*	*Actb*
**7 days**	*Ppia*	*Rpl13a*	*Ywhaz*	*Hprt1*	*B2m*	*Pgk1*	*Sdha*	*Actb*	*Gapdh*
**Overall**	***Pgk1***	***Ppia***	***Rpl13a***	***Ywhaz***	***Hprt1***	***B2m***	***Sdha***	***Gapdh***	***Actb***

Figure S9. The reference gene stability within the entorhinal cortex of control and PTZ-treated rats at different time points after seizures. Seizures was induced at P21. Gene expression stability in PTZ-treated and vehicle control animals at different time points (3 h, 1, 3, 7 days; n – 4-7 per group) was assessed by RefFinder online tool.

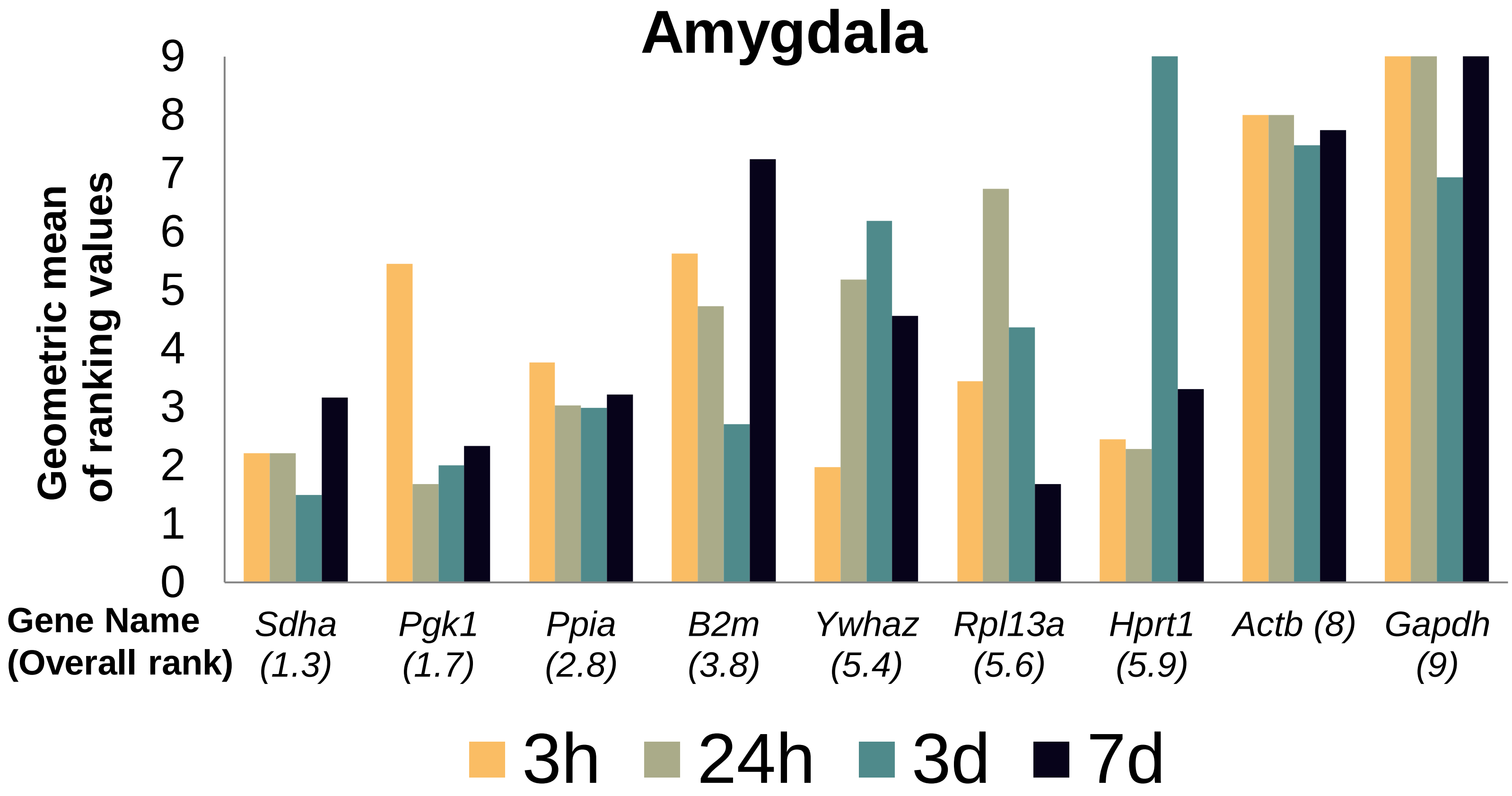

Time after Seizures	RefFinder Comprehensive ranking (Most stable→Least stable)								
**3 h**	*Ywhaz*	*Sdha*	*Hprt1*	*Rpl13a*	*Ppia*	*Pgk1*	*B2m*	*Actb*	*Gapdh*
**24 h**	*Pgk1*	*Sdha*	*Hprt1*	*Ppia*	*B2m*	*Ywhaz*	*Rpl13a*	*Actb*	*Gapdh*
**3 days**	*Sdha*	*Pgk1*	*B2m*	*Ppia*	*Rpl13a*	*Ywhaz*	*Gapdh*	*Actb*	*Hprt1*
**7 days**	*Rpl13a*	*Pgk1*	*Sdha*	*Ppia*	*Hprt1*	*Ywhaz*	*B2m*	*Actb*	*Gapdh*
**Overall**	***Sdha***	***Pgk1***	***Ppia***	***B2m***	***Ywhaz***	***Rpl13a***	***Hprt1***	***Actb***	***Gapdh***

Figure S10. The reference gene stability within the amygdala of control and PTZ-treated rats at different time points after seizures. Seizures was induced at P21. Gene expression stability in PTZ-treated and vehicle control animals at different time points (3 h, 1, 3, 7 days; n – 4-7 per group) was assessed by RefFinder online tool.

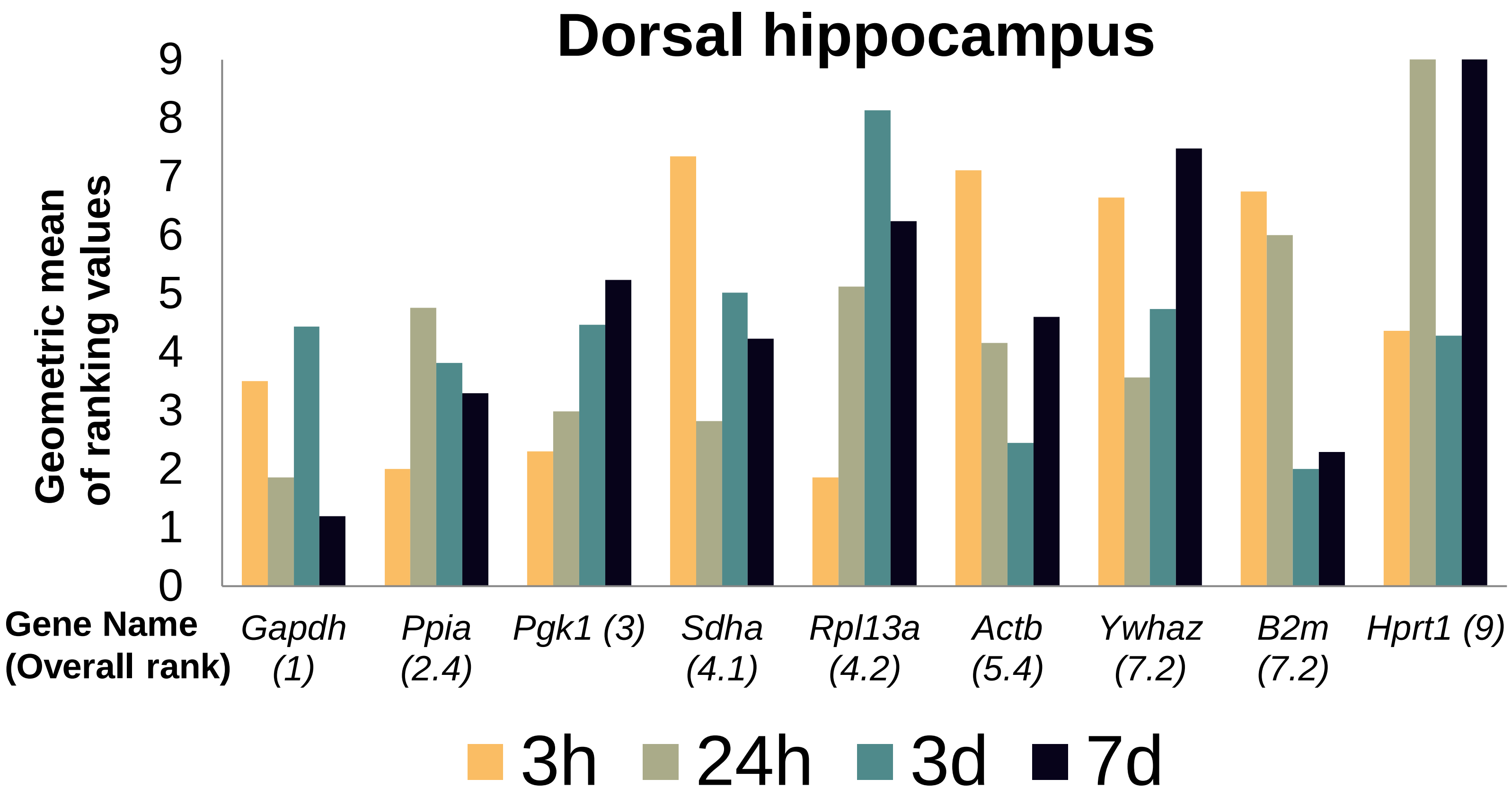

Time after Seizures	RefFinder Comprehensive ranking (Most stable→Least stable)								
3 h	*Rpl13a*	*Ppia*	*Pgk1*	*Gapdh*	*Hprt1*	*Ywhaz*	*B2m*	*Actb*	*Sdha*
24 h	*Gapdh*	*Sdha*	*Pgk1*	*Ywhaz*	*Actb*	*Ppia*	*Rpl13a*	*B2m*	*Hprt1*
3 days	*B2m*	*Actin*	*Ppia*	*Hprt1*	*Gapdh*	*Pgk1*	*Ywhaz*	*Sdha*	*Rpl13a*
7 days	*Gapdh*	*B2m*	*Ppia*	*Sdha*	*Actin*	*Pgk1*	*Rpl13a*	*Ywhaz*	*Hprt1*
**Overall**	***Ppia***	***Rpl13a***	***Sdha***	***Pgk1***	***B2m***	***Actb***	***Gapdh***	***Ywhaz***	***Hprt1***

Figure S11. The reference gene stability within the dorsal hippocampus of control and PTZ-treated rats at different time points after seizures. Seizures was induced at P21. Gene expression stability in PTZ-treated and vehicle control animals at different time points (3 h, 1, 3, 7 days; n – 4-7 per group) was assessed by RefFinder online tool.

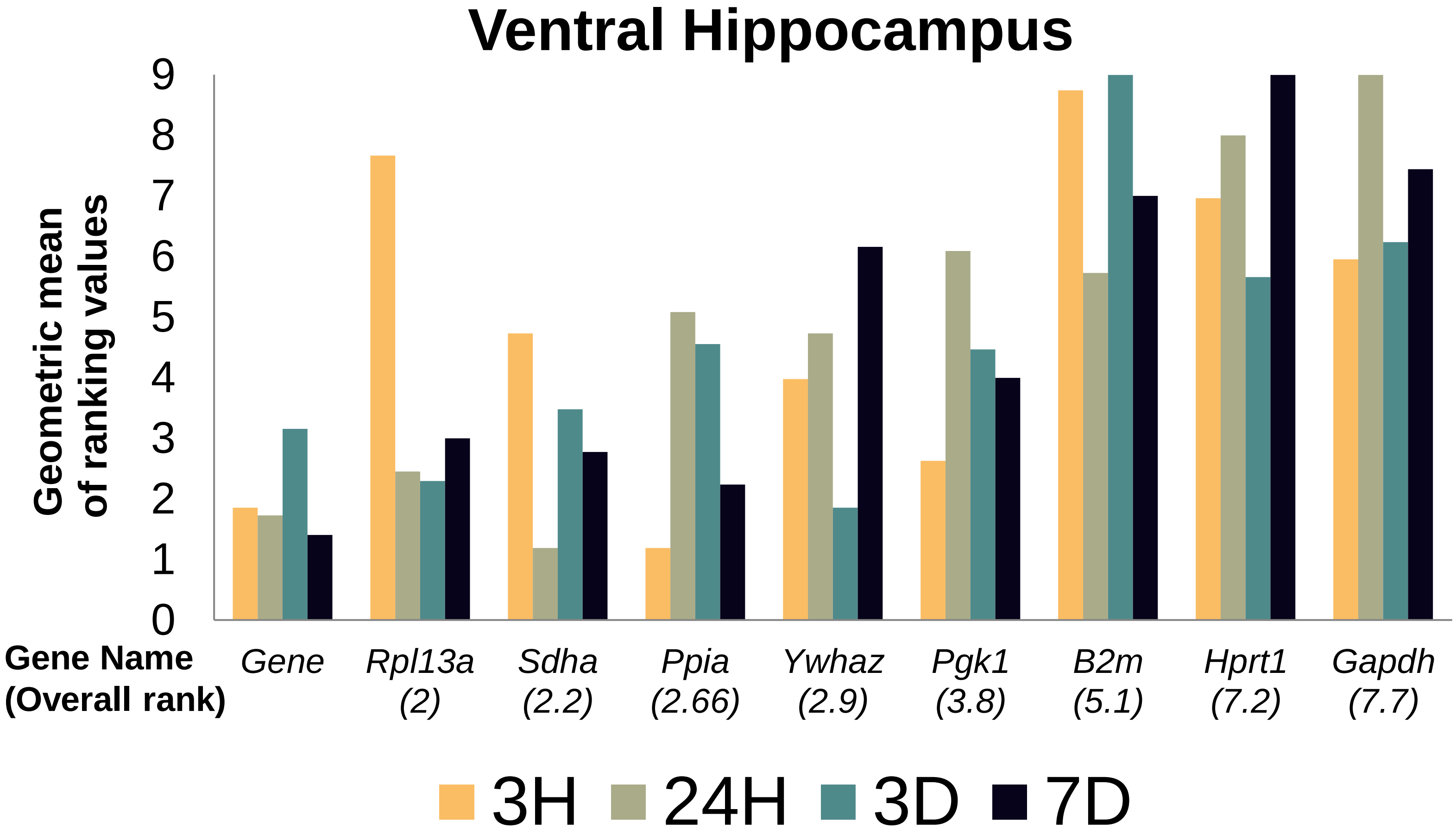

Time after Seizures	RefFinder Comprehensive ranking (Most stable→Least stable)								
**3 h**	*Ywhaz*	*Rpl13a*	*B2m*	*Pgk1*	*Ppia*	*Actb*	*Gapdh*	*Sdha*	*Hprt1*
**24 h**	*Ppia*	*Rpl13a*	*Sdha*	*Pgk1*	*Ywhaz*	*Hprt1*	*B2m*	*Gapdh*	*Actb*
**3 days**	*Pgk1*	*Sdha*	*Rpl13a*	*Ppia*	*B2m*	*Ywhaz*	*Gapdh*	*Actb*	*Hprt1*
**7 days**	*Rpl13a*	*Ywhaz*	*Ppia*	*Sdha*	*B2m*	*Pgk1*	*Hprt1*	*Actb*	*Gapdh*
**Overall**	***Ppia***	***Rpl13a***	***Sdha***	***Pgk1***	***B2m***	***Actb***	***Gapdh***	***Ywhaz***	***Hprt1***

Figure S12. The reference gene stability within the ventral hippocampus of control and PTZ-treated rats at different time points after seizures. Seizures was induced at P21. Gene expression stability in PTZ-treated and vehicle control animals at different time points (3 h, 1, 3, 7 days; n – 4-7 per group) was assessed by RefFinder online tool.
